# The Effects of Prenatal Iron Supplementation on Offspring Neurodevelopment in Upper Middle- or High-Income Countries: A Systematic Review

**DOI:** 10.3390/nu16152499

**Published:** 2024-07-31

**Authors:** Najma A. Moumin, Emily Shepherd, Kai Liu, Maria Makrides, Jacqueline F. Gould, Tim J. Green, Luke E. Grzeskowiak

**Affiliations:** 1 Women and Kids, South Australian Health and Medical Research Institute, Adelaide, SA 5000, Australia; najma.moumin@sahmri.com (N.A.M.); emily.shepherd@sahmri.com (E.S.); maria.makrides@sahmri.com (M.M.); jacqueline.gould@sahmri.com (J.F.G.); tgreen@flinders.edu.au (T.J.G.); 2 Discipline of Pediatrics, Adelaide Medical School, The University of Adelaide, Adelaide, SA 5005, Australia; 3 Discipline of Obstetrics and Gynecology, Adelaide Medical School, The University of Adelaide, Adelaide, SA 5005, Australia; kai.liu@adelaide.edu.au; 4 Lifelong Health, South Australian Health and Medical Research Institute, Adelaide, SA 5000, Australia; 5 College of Nursing and Allied Health, Caring Futures Institute, Flinders University, Adelaide, SA 5042, Australia; 6 College of Medicine and Public Health, Flinders Health and Medical Research Institute, Flinders University, Adelaide, SA 5042, Australia

**Keywords:** iron supplementation, neurodevelopment, pregnancy, prenatal

## Abstract

Iron supplementation is commonly recommended for the prevention and treatment of maternal iron deficiency (ID) or iron deficiency anemia (IDA). However, the impacts of prophylactic of therapeutic prenatal iron supplementation on child neurodevelopment in upper middle-income (UMI) and high-income countries (HICs), where broad nutritional deficiencies are less common, are unclear. To investigate this, we conducted a systematic review, searching four databases (Medline, CINAHL, EMBASE, Cochrane Library) through 1 May 2023. Randomized controlled trials (RCTs) assessing oral or intravenous iron supplementation in pregnant women reporting on child neurodevelopment (primary outcome: age-standardized cognitive scores) were eligible. We included three RCTs (five publications) from two HICs (Spain and Australia) (*N* = 935 children; *N* = 1397 mothers). Due to clinical heterogeneity of the RCTs, meta-analyses were not appropriate; findings were narratively synthesized. In non-anemic pregnant women, prenatal iron for prevention of IDA resulted in little to no difference in cognition at 40 days post-partum (1 RCT, 503 infants; very low certainty evidence). Similarly, the effect on the intelligence quotient at four years was very uncertain (2 RCTs, 509 children, very low certainty evidence). No RCTs for treatment of ID assessed offspring cognition. The effects on secondary outcomes related to language and motor development, or other measures of cognitive function, were unclear, except for one prevention-focused RCT (302 children), which reported possible harm for children’s behavioral and emotional functioning at four years. There is no evidence from UMI countries and insufficient evidence from HICs to support or refute benefits or harms of prophylactic or therapeutic prenatal iron supplementation on child neurodevelopment.

## 1. Introduction

Iron requirements increase substantially during pregnancy to facilitate maternal blood volume expansion and fetal iron transfer, placing women at risk of iron deficiency (ID) with or without anemia [1,2]. A 2019 Lancet Global Health report noted that approximately 36% of pregnant women (15–49 years) are anemic, and ID is responsible for one quarter to one half of all cases worldwide [2]. Although low- and middle-income countries (LMICs) are disproportionately affected, the prevalence of iron deficiency anemia (IDA) among pregnant women is as high as 15% in some high-income countries (HICs) [2]; 28 to 85% of European women have been estimated to be ID in the third trimester of pregnancy, the peak of fetal iron transfer [3].

Oral iron supplementation is recognized as a first-line therapy to correct maternal ID and IDA [4,5,6,7,8,9,10]; however, routine supplementation to prevent ID is not recommended in most HICs [4,5,6,9,11] due to limited evidence of clinical benefits for maternal and child health outcomes. Despite this, prenatal multivitamins containing up to 60 mg of elemental iron are commonly consumed by women, irrespective of iron status [12]. The implications for child neurodevelopment are unknown. Evidence surrounding the optimal oral dose for treating IDA is also unclear, with recommendations varying from 40 to 200 mg of elemental iron between countries [13,14,15,16].

Observational studies have long reported associations between maternal ID and poor cognitive outcomes in children [17,18,19]. However, recent evidence from a large Dutch cohort study (*N* = 2479 mother–child dyads) has also linked high maternal iron status in early pregnancy with both a lower IQ and a smaller brain size in children at six and ten years, respectively [20]. In the study, although one third of women with high serum ferritin in early pregnancy reported prenatal multivitamin use, it is unclear what dose of iron, if any, these contained [20]. Previous systematic reviews and meta-analyses evaluating prenatal iron supplementation and childhood neurodevelopment were unable to draw definitive conclusions, including due to the limited number of studies at the time and a focus on prophylactic iron supplementation (rather than all forms of treatment) [21,22]. They were also strongly influenced by data from LMICs [21], and as such, their findings were unlikely to be generalizable to high-resource settings, where nutritional deficiencies are less prevalent. A contemporary evaluation of the impacts of prenatal iron supplementation for the prevention and/or treatment of IDA on child neurodevelopment in UMI and HICs is therefore necessary, especially given the ubiquity of iron-containing prenatal supplements in these settings.

## 2. Materials and Methods

This systematic review was prepared in accordance with the Preferred Reporting Items for Systematic Reviews and Meta-Analyses (PRISMA) checklist [23] (Table S1) and was registered with the International Prospective Register of Systematic Reviews [24] (CRD42023429580).

### 2.1. Data Sources and Search Strategy

We consulted an experienced research librarian to develop the search strategy using combinations of controlled vocabulary (such as MeSH) and free text words (Table S2). We then performed comprehensive searches across four main databases (Medline, CINAHL, EMBASE, Cochrane Library) through 1 May 2023. Additional manual searches on Google Scholar for recent RCTs were also completed. No date or language restrictions were applied; however, because of logistical constraints, for non-English papers, only those with an available English full-text translation were retrieved.

### 2.2. Eligiblity Criteria and Study Selection

We included RCTs (individual or cluster-randomized) where the intervention occurred in pregnant women living in upper middle-income (UMI) or HICs as defined by the World Bank gross national income per capita at the time of the study [25]. RCTs were eligible if: (1) women received oral or intravenous (IV) iron in pregnancy; (2) the comparator group received placebo or no intervention, iron via a different dose or for a different duration, or iron with a co-intervention where the quantities of other nutrients were equal across treatment groups; and (3) offspring neurodevelopmental outcomes were reported. Our primary outcome was the global cognition or intelligence quotient (IQ), where a psychometric test provided an age-standardized score (mean = 100, standard deviation (SD) = 15). Secondary outcomes included other measures of neurodevelopment, such as other aspects of cognitive functioning, language, motor skills, academic abilities, and emotional and behavioral functioning. Studies were excluded if they were quasi-randomized, cross-over and non-RCTs, cohort studies, case-control studies, cross-sectional studies, case series, and case reports. Conference abstracts were excluded.

Retrieved publications were uploaded into Covidence [26] for duplicate removal and screening. Two independent reviewers (NAM and TJG) completed title and abstract screening. The same reviewers assessed full-text articles for inclusion.

### 2.3. Data Extraction and Quality Appraisal

Data were extracted using a standardized form, piloted by NAM and KL, and reviewed by ES. NAM and KL completed data extraction independently; any discrepancies were resolved through discussion with JFG. Where outcome data were missing or data conversions were needed, we contacted study authors.

The quality appraisal of included RCTs was conducted independently by NAM and KL using established guidelines from the Cochrane Handbook for Systematic Reviews of Interventions [27]. The certainty of the evidence was appraised following the Grading of Recommendations, Assessment, Development and Evaluation (GRADE) approach [28] for our primary outcome.

### 2.4. Data Synthesis

Due to expected variations in assessment tools, we planned to perform random-effects meta-analyses using Review Manager, Version 5.4 [29] to calculate standardized mean differences with 95% confidence intervals (CI) for primary and secondary outcomes according to child age at assessment: ≤12 months, 1–3 years, and 4–8 years.

We planned to conduct subgroup analyses according to participant (maternal iron and hemoglobin status) and treatment (timing, dose, and duration) characteristics. Sensitivity analyses excluding studies with a high risk of bias were also planned.

## 3. Results

### 3.1. Search Results and Trial Characteristics

**Figure 1:**
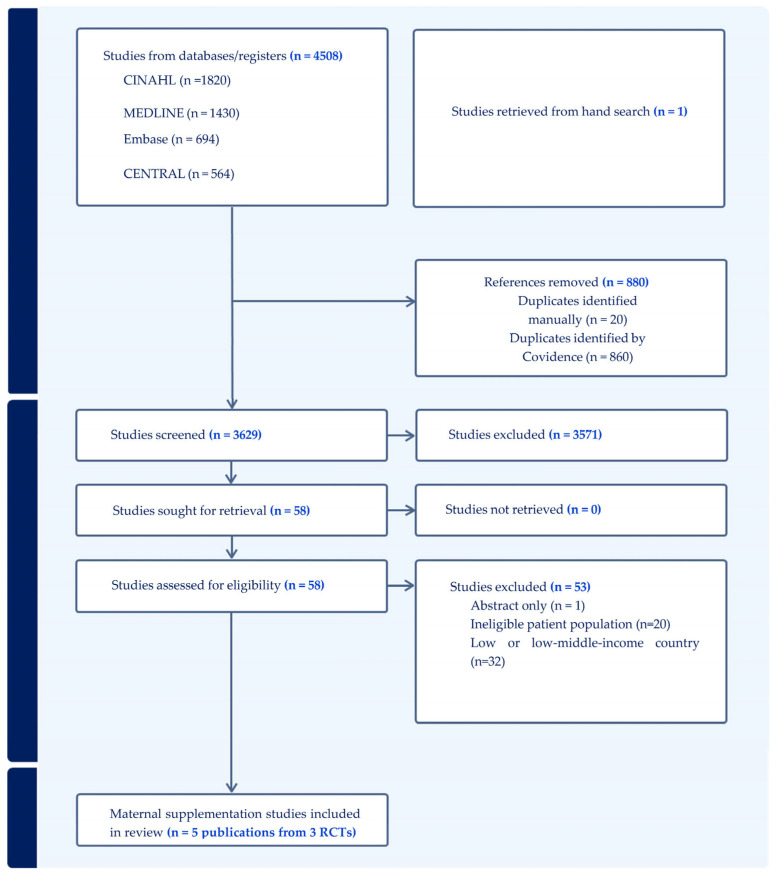
Study flow diagram.

Our initial search identified 4509 articles, 880 of which were duplicates. After screening 3629 articles, 58 were assessed in full for inclusion, and five (relating to three RCTs) were included in this review (Figure 1). Of those excluded, 32 reported on studies conducted in LMICs, 20 did not include the population of interest (one animal and 19 child supplementation RCTs), and one RCT was reported as a conference abstract only. A list of the excluded studies is provided in Table S3.

### 3.2. Study Characteristics

A summary of the included RCTs is provided in Table 1. Five publications reported findings from three prenatal iron supplementation RCTs in two high-income countries, Australia [30,31,32] and Spain [33,34]. Additional data were sought from the authors of two RCTs [30,33,34], and both provided the information requested.

Two of the RCTs [31,32,33,34] supplemented non-anemic women (hemoglobin > 110 g/L) with oral iron from their first or second trimester until delivery for the prevention of IDA; however, there were differences in their comparators. In the AMBIT RCT (Refs. [31,32]), women received 20 mg of oral iron or placebo. In comparison, in the ECLIPSES RCT [33,34], women with normal hemoglobin (110–130 g/L) were randomized to either 80 or 40 mg iron daily (Stratum 1) and those with high hemoglobin (>130 g/L) to either 40 or 20 mg iron daily (Stratum 2). The third RCT (IV Iron RCT) compared two doses (500 or 1000 mg) of IV ferric carboxymaltose (FCM) for treating ID defined as serum ferritin (SF) < 15 µg/mL or SF < 50 µg/mL and transferrin saturation <20% with elevated c-reactive protein in the second or third trimester [30].

Child neurodevelopment was reported as a secondary outcome in the three RCTs and only one was adequately powered to assess this outcome [32]. Measures of neurodevelopment varied between RCTs, as did the instruments used and the ages of the children at assessment (see Table 1). Some assessments were administered by psychologists or research assistants, whilst others were parent- or teacher-completed questionnaires. Evaluated outcomes included various measures of cognitive, language, and motor development, and emotional and behavioral functioning.

### 3.3. Risk of Bias

Table 2 provides a summary of the risk of bias for the included RCTs (with further details in Table S4). All were at high risk of attrition bias due to incomplete outcome data > 20% [27]. All had an unclear or high risk of reporting bias due to lack of clear outcome pre-specification.

### 3.4. Effect of Prenatal Iron Supplementation on Primary Outcome: Age-Standardized Cognitive Score or Intelligence Quotient

Only one prevention RCT (ECLIPSES) measured cognition in infancy using the Bayley-III at ~40 days post-partum [33]. Very low certainty evidence suggested that higher- versus lower-dose prenatal iron did not benefit or harm cognitive development in infants < 12 months (80 mg versus 40 mg oral iron: MD −0.98, 95% CI −2.87, 0.91, 328 infants; 40 mg versus 20 mg oral iron: MD 2.00, 95% CI −0.69, 4.69, 175 infants) (Table 3).

Both prevention RCTs (AMBIT [32] and ECLIPSES [34]) measured offspring IQ at four years using the Stanford–Binet Intelligence Scale (SBIS) and the Wechsler Preschool and Primary Scale of Intelligence Version 4 (WPPSI-IV), respectively. Very low-certainty evidence suggested that neither higher versus lower dose iron (80 mg versus 40 mg oral iron: MD 0.57; 95% CI −3.00, 4.14, 182 children; 40 mg vs. 20 mg oral iron: MD 0.77, 95% CI −3.30, 4.84, 106 children) nor iron versus placebo (MD 0.00, 95% CI −2.48, 2.48, 302 children) improved or diminished IQ at four years (Table 3).

There was no age-standardized assessment of child cognition in the RCT evaluating the treatment of ID.

### 3.5. Effect of Prenatal Iron Supplementation on Secondary Outcomes

#### 3.5.1. Language: Prevention of IDA

There were no clear differences in language or subscales of language (expressive and receptive language) scores (Bayley-III) at 40 days between children born to women receiving higher versus lower doses of iron in both strata of the ECLIPSES RCT. The proportion of infants showing signs of developmental delays (score < 85) was similar between the groups.

At the four-year follow-up of the AMBIT RCT, there were no clear differences in the verbal reasoning scores (SBIS) of children born to women receiving iron or placebo. Similarly, in the ECLIPSES RCT four-year follow-up, there were no clear differences between the treatment groups in both strata in any subscales of IQ related to language (verbal comprehension index, vocabulary acquisition index, non-verbal index) (WPPSI-IV) in intention-to-treat analyses (unpublished data) (Table S5). However, per-protocol analyses reported significant differences in subscales of the IQ test according to maternal serum ferritin status at entry [34]. Children whose mothers received 80 mg of iron but entered pregnancy with serum ferritin > 65 µg/L had lower verbal comprehension index and vocabulary acquisition index scores; there were no differences between intervention and control children of women with low (<15 µg/L) or normal (15–65 µg/L) serum ferritin in early pregnancy in either stratum.

#### 3.5.2. Language: Treatment of ID

There were no clear differences in communication scores between children of women treated for ID with 1000 versus 500 mg of IV FCM (Ages and Stages Questionnaire (ASQ)) at 12 months (Table S5).

#### 3.5.3. Motor Development: Prevention of IDA

There were no clear differences in motor or subscales of motor development (Bayley-III) at 40 days between children born to women receiving higher versus lower iron doses from both strata in the ECLIPSES RCT. The proportion of infants showing signs of developmental delays (score < 85 for main scale or <7 for subscales) was also similar between the groups.

#### 3.5.4. Motor Development: Treatment of ID

Likewise, there were no clear differences between children born to mothers receiving higher versus lower doses of iron in gross or fine motor scores at 12 months (ASQ) in the IV Iron RCT.

#### 3.5.5. Child Emotional and Behavioral Functioning: Prevention of IDA

Both RCTs assessing behavior in older children measured aspects of behavior related to emotion. In the AMBIT RCT follow-up, there were no clear differences in the total behavioral difficulties mean score or any sub-domains (Strengths and Difficulties Questionnaire (SDQ)) between the children born to women receiving iron versus placebo at the four or six to eight year follow up [31,32]. However, more children born to women who received iron had a total difficulties score ≥ 17 (indicating abnormal behavior) at four years of age. Although this effect was not present at six to eight years of age, abnormal scores for teacher-rated peer problems were higher in the iron versus placebo group (RR 3.70, 95% CI 1.06, 12.91). There were no clear differences between the groups in the mean scores for child temperament measured (Short Temperament Scale for Children (STS)) at the six to eight-year follow-up or the percentage of children with difficult temperament (>1 SD above the mean).

In the four-year follow-up of the ECLIPSES RCT, there were no differences in emotion recognition (Developmental Neuropsychological Assessment (NEPSY-II)) in either stratum in intent-to-treat analyses (unpublished data). However, reported per-protocol analyses stratified by the maternal serum ferritin status at RCT entry showed higher emotion recognition scores in children of women supplemented with 80 mg iron who entered pregnancy with normal hemoglobin of 110–130 g/L and serum ferritin < 15 µg/L compared with children of women supplemented with 40 mg iron [34]. Conversely, children of women supplemented with 40 mg iron who entered pregnancy with high hemoglobin > 130 g/L and serum ferritin > 65 µg/L scored lower on emotion recognition than children of women supplemented with 20 mg.

#### 3.5.6. Child Emotional and Behavioral Functioning: Treatment of ID

There were no clear differences between children born to women who received higher versus lower doses of iron in personal–social development scores (ASQ) at 12 months in the IV Iron RCT (unpublished data).

#### 3.5.7. Other Cognitive Outcomes: Prevention of IDA

Other outcomes relating to memory, processing speed, and visual and quantitative reasoning were assessed in the AMBIT RCT and ECLIPSES RCT at the four-year follow-up [32,34]. Neither RCT showed any clear differences between groups for these outcomes (Table S5).

## 4. Discussion

We screened 3629 articles and ultimately included three RCTs with 935 children from two HICs, Spain and Australia. No RCTs from UMI countries were identified. Meta-analyses were not possible due to the small number of clinically heterogeneous RCTs. The quality of evidence was very low, and all three RCTs had a high risk of bias in the incomplete outcome domain. Considering the two RCTs focused on the prevention of IDA, prenatal iron supplementation versus placebo, and higher versus lower doses of iron showed no clear evidence of benefit or harm on age-standardized cognitive scores or IQ. Across the three RCTs (assessing the prevention of IDA and treatment of ID), there were similarly little to no effects on language, motor development, child emotional and behavioral functioning, or other aspects of cognition related to memory, processing speed, and visual or quantitative reasoning.

Child cognitive outcomes are among many clinical outcomes necessary to consider when balancing the risks and benefits of iron supplementation in pregnancy. A 2015 Cochrane review evaluated the impact of preventive oral iron on several maternal and neonatal health outcomes including maternal ID, anemia, death, infection during pregnancy, low birthweight, preterm birth, neonatal death, and congenital anomalies. Apart from hematological improvements for the mother at term, beneficial effects on other clinical outcomes were equivocal [7]. In LMICs, where the prevalence of IDA is high, the World Health Organization recommends that pregnant women take 30–60 mg of oral iron from early pregnancy until delivery [10]. However, in HICs, prophylactic iron use in pregnancy is not routinely recommended, due to the potential risks of iron overload [35,36,37]. In these settings, tailored recommendations based on the women’s iron status may be preferable [37].

Lending support for caution with routine supplementation, evidence from the two prevention-focused RCTs in our review suggests potential harms to childhood emotional and behavioral functioning [31,32] and subscales of intelligence related to language and memory [34] at four years. Care is required with the interpretation of these findings due to RCT limitations. Despite receiving 20 mg of oral iron from 20 weeks’ gestation through delivery and reporting a high compliance of 86%, one third of intervention women were ID at delivery in the AMBIT RCT [32], raising questions about adherence and whether this potentially influenced the magnitude of effect. Furthermore, sub-group analyses according to maternal ferritin in the ECLIPSES RCT were adjusted for maternal iron status late in pregnancy after women already received the intervention, potentially introducing bias into the causal pathway [34]. Thus, the impact of prophylactic prenatal iron on child neurodevelopment in high-resource settings remains uncertain.

Similarly, there was insufficient evidence to determine the impact of different doses of iron for the treatment of established ID on child neurodevelopment. The IV Iron RCT was powered on the proportion of participants who required a repeat iron infusion to determine the superiority of one dose over another for correcting ID [30]. Although both groups received the same dose (500 mg IV FCM) for the repeat infusion, women in the 500 mg group were over two times more likely to require a repeat infusion compared with women in the 1000 mg group (RR 2.05, 95% CI 1.45–2.91; *p* < 0.001) [30], which may have masked any potential differences in ASQ scores attributable to the different doses of iron. The lack of studies comparing long-term neurodevelopmental outcomes with IV or oral iron is also notable, particularly given the rapid increase in use (and cost) of IV iron in this population, and the lack of evidence supporting improvement in maternal or infant clinical outcomes [8,38].

All five publications reviewed had a high risk of bias in the incomplete outcome reporting domain due to significant loss to follow-up (30–66%). Furthermore, only one publication included a sample size calculation and was adequately powered to detect an effect on IQ [32]. The remainder either did not report a sample size estimate for the outcomes measured or were powered on a different primary outcome altogether [30,33,34]. Although intent-to-treat analyses were requested for both follow-up studies from the ECLIPSES RCT [33,34], data were only provided for the four-year follow-up (unpublished). While the Bayley-III assessment at 40 days may be useful for assessing signs of major disabilities, cognitive abilities are not well-developed or measurable at this age and results of this outcome may not be generalizable. Finally, both parents and teachers who completed outcome assessments were unblinded in the follow-up at six to eight years in the AMBIT RCT [31].

To our knowledge, this is the first systematic review assessing the effects of prenatal iron supplementation on child neurodevelopment in HICs in the context of the prevention of IDA and treatment of ID. Our review includes two new RCTs that were not included in previous systematic reviews and meta-analyses [21,22]. We planned to examine the effect of baseline hemoglobin and iron status, dose, duration, and the timing of iron supplementation in pregnancy on offspring neurodevelopment; however, this was not possible due to a lack of data. The included RCTs were clinically heterogenous, precluding meta-analysis, and making it difficult to draw definitive conclusions. Despite uncertainty in the findings, the possibility of harm to children born to iron-replete women who received further supplementation highlights the urgent need for adequately powered RCTs to determine the safety of routine supplement use in high-resource settings.

## 5. Conclusions

Very low-certainty evidence suggests that prenatal iron compared with a placebo or a high versus low dose for the prevention of IDA may not confer harm or benefit on child neurodevelopment in high-resource settings. The effect on cognition is unknown and the certainty of evidence for other aspects of neurodevelopment is very low for higher versus lower doses of prenatal iron for the treatment of ID. High-quality well-powered RCTs are required to determine the impacts of routine iron supplementation for preventing ID and of different doses of iron for treatment of ID on child neurodevelopmental outcomes in both UMI and HICs.

## Figures and Tables

**Table 1:** Characteristics of included RCTs.

Citation		Population Enrolled, Location	Sample Size Enrolled	Intervention Arms (Type, Dose, Frequency)	Duration of Intervention	Outcome, Instrument Used, Age Assessed	Children Assessed
**Prevention of IDA**							
AMBIT RCT	Zhou 2006 [32]	Non anaemic (Hb > 110 g/L) pregnant women with unknown iron status at 20 wks gestation, Australia	n = 216 intervention, n = 214 placebo	*Intervention*: 20 mg oral iron, once daily *Control*: Placebo, once daily	20 wks gestation until delivery	4 years: IQ, behavior using SBIS and SDQ 6–8 years: behavior using SDQ and STS for children	4 years [32] *Intervention*: IQ (n = 153), behavior (n = 151) *Control*: IQ (n = 149), behavior (n = 149) 6–8 years [31] *Intervention*: parent-rated SDQ (n = 132), teacher-rated SDQ (n = 112), parent-rated STS (n = 132) *Control*: parent-rated SDQ (n = 132), teacher-rated SDQ (n = 113), parent-rated STS (n = 132)
	Parsons 2008 [31]						
ECLIPSES RCT	Iglesias-Vazquez 2022 [33]	Non anaemic (Hb > 110 g/L) pregnant women with unknown iron status, ≤12 wks gestation, Spain	Stratum 1 (Hb 110–130 g/L): n = 268 intervention, n = 261 control	*Stratum 1* *Intervention*: 80 mg oral iron, daily *Control*: 40 mg oral iron, daily	12 wks gestation until delivery	40 days: cognitive, motor, and language development using Bayley-III 4 years: IQ using WPPSI-IV and NEPSY-II	40 days [33] *Intervention*: Stratum 1 (n = 161), Stratum 2 (n = 93) *Control*: Stratum 1 (n = 167) Stratum 2 (n = 82)
	Iglesias-Vazquez 2023 [34]		Stratum 2 (Hb > 130 g/L): n = 132 intervention, n = 130 control	*Stratum 2* *Intervention*: 40 mg oral iron, daily *Control*: 20 mg oral iron, daily			4 years [34] *Intervention*: Stratum 1 (n = 92), Stratum 2 (n = 55) *Control*: Stratum 1 (n = 90) Stratum 2 (n = 51)
**Treatment of ID**							
IV Iron RCT	Froessler 2023 [30]	ID (SF < 15 µg/mL or SF < 50 µg/mL and TSAT < 20% with elevated CRP) pregnant women in the second or third trimester, Australia	n = 139 intervention, n = 165 control	*Intervention*: 1000 mg IV FCM, single dose *Control*: 500 mg IV FCM, single dose	Once	12 months: communication, gross motor, fine motor, problem solving, personal–social development using ASQ	12 months [30] *Intervention*: ASQ (n = 53) *Control*: ASQ (n = 75–77)

Abbreviations: ASQ: Ages and Stages Questionnaire; Bayley-III: Bayley Scales of Infant Development version 3; CRP: c-reactive protein; FCM: ferric carboxymaltose; Hb: haemoglobin; ID: iron deficiency; IDA: iron deficiency anaemia; IQ: intelligence quotient; IV: intravenous; NEPSY-II: Neuropsychological Assessment second edition; RCT: randomized controlled trial; SBIS: Stanford–Binet Intelligence Scale; SDQ: Strengths and Difficulties Questionnaire; SF: serum ferritin; STS: Short Temperament Scale; TSAT: transferrin saturation; wks: weeks; WPPSI-IV: Wechsler Preschool and Primary Scale of Intelligence version 4.

**Table 2:** Risk of bias.

Author, Year		Selection Bias (Random Sequence Generation)	Selection Bias (Allocation Concealment)	Performance Bias (Blinding of Participants and Personnel)	Detection Bias (Blinding of Outcome Assessment)	Attrition Bias (Incomplete Outcome Data)	Reporting Bias (Selective Outcome Reporting)	Other Bias
**Prevention of IDA**								
AMBIT RCT	Zhou 2006 [32]	Low risk	Low risk	Low risk	Low risk	High risk	Unclear risk	Low risk
	Parsons 2008 [31]			High risk	High risk			
ECLIPSES RCT	Iglesias-Vazquez 2022 [33]	Low risk	Low risk	Low risk	Low risk	High risk	Low risk	High risk ^a^
	Iglesias-Vazquez 2023 [34]						Unclear risk	High risk ^b^
**Treatment of ID**								
IV Iron RCT	Froessler 2022 [30]	Low risk	Low risk	Low risk	Low risk	High risk	High risk	Low risk

Abbreviations: ID: iron deficiency; IDA: iron deficiency anaemia; RCT: randomized controlled trial. ^a^ Intent to treat analyses requested but were not available. Results are per-protocol. ^b^ Intent to treat analyses requested and provided.

**Table 3:** Summary of results: primary outcomes.

Primary Outcome	Number of Participants (RCTs)	MD (95% CI)	Quality of Evidence (GRADE)
**Oral iron for prevention of IDA**			
**Global cognition infants at 40 days (Bayley-III)**			
Baseline Hb 110–130 g/L			
80 mg versus 40 mg oral iron	328 (1 RCT) [33]	−0.98 (−2.87, 0.91)	⨁◯◯◯ ^a,b^ Very Low
Baseline Hb > 130 g/L			
40 mg versus 20 mg oral iron	175 (1 RCT) [33]	2.00 (−0.69, 4.69)	⨁◯◯◯ ^a,b^ Very Low
**Intelligence quotient 4 years (SBIS or WPPSI-IV)**			
Baseline Hb 110–130 g/L			
80 mg versus 40 mg oral iron	182 (1 RCT) [34]	0.57 (−3.00, 4.14)	⨁◯◯◯ ^a,c^ Very low
Baseline Hb > 130 g/L			
40 mg versus 20 mg oral iron	106 (1 RCT) [34]	0.77 (−3.30, 4.84)	⨁◯◯◯ ^a,c^ Very low
Baseline Hb > 110 g/L			
20 mg oral iron versus placebo	302 (1 RCT) [32]	0.00 (−2.48, 2.48)	⨁◯◯◯ ^a,d^ Very Low

Note: GRADE Working Group grades of evidence. Very low certainty: we have very little confidence in the effect estimate: the true effect is likely to be substantially different from the estimate of effect. Abbreviations: Bayley-III: Bayley Scales of Infant Development version 3; CI: confidence interval; GRADE: Grading of Recommendations, Assessment, Development and Evaluation; g/L: gram per liter; Hb: haemoglobin; IDA: iron deficiency anaemia; MD: mean difference; RCT: randomized controlled trial; SBIS: Stanford–Binet Intelligence Scale; WPPSI-IV: Wechsler Preschool and Primary Scale of Intelligence version 4. ^a^ Downgraded 1 level for imprecision: wide confidence interval crossing line of no effect including potentially harmful or beneficial effect. ^b^ Downgraded 2 levels for risk of bias: attrition ~40% and per-protocol analysis. ^c^ Downgraded 2 levels for risk of bias: attrition ~60% and unclear risk of selective outcome reporting. ^d^ Downgraded 2 levels for risk of bias: attrition ~30% and unclear risk of selective outcome reporting.

## Supplementary Materials

The following supporting information can be downloaded at: https://www.mdpi.com/article/10.3390/nu16152499/s1: Table S1: Prisma checklist; Table S2: Search terms; Table S3: Excluded studies; Table S4: Risk of bias rationale; Table S5: Secondary outcomes.
