# Supplementary material for: The Effects of Prenatal Iron Supplementation on Offspring Neurodevelopment in Upper Middle- or High-Income Countries: A Systematic Review
Source: Nutrients. 2024 Jul 31;16(15):2499. doi: 10.3390/nu16152499 (PMC11314290; doi:10.3390/nu16152499)
Supplement: Supplementary file 1 [file nutrients-16-02499-s001.zip › Table S2 Search Terms.pdf]

Table S2. Search Strategy

Medline

**Supplement or IV iron**	**Population**	**Cognitive outcomes**
(iron.sh AND dietary supplements.sh)<br>OR (iron.sh AND exp fortified food)<br>OR iron fortified formula.mp<br><br>OR ((iron* OR ferrous* OR ferric* OR micronutrient*) adj4 (supplement* OR compound* OR therap* OR powder OR sprinkles OR syrup* OR drops* OR tablet*OR treatment* OR formula*)).mp<br><br>OR ((IV OR intravenous OR injection OR transfusion) adj4 (Iron dextran OR iron-dextran complex OR Iron sucrose OR Sodium ferric gluconate OR Ferumoxytol OR Ferric carboxymaltose OR FCM OR Iron isomaltoside OR ferric derisomaltose OR Iron polymaltose OR Iron saccharate)).mp	exp Pregnancy OR (pregnan* OR utero OR maternal OR antenatal OR gestat* OR f?etal OR f?etus).mp OR Child, preschool.sh OR exp infant OR exp Infant, Premature/ OR (toddler* OR neonat* OR perinat* OR newborn* OR early childhood OR offspring OR infan* OR baby OR babies).mp	exp brain OR<br>exp child development OR<br>exp psychomotor performance OR exp speech OR exp intelligence tests OR comprehension.sh OR<br>exp cognition OR exp intelligence OR exp memory OR<br>exp attention OR exp visual acuity<br><br>OR (neur* OR cogni* OR behavi* OR emoti* OR socioemot* OR socio-emot* OR language* OR linguistic* OR spatial learn* OR spatial navigation* OR spatial processing* OR motor* OR learn* OR mental* OR Bayley Scales* OR child development OR psychomotor OR speech OR brain OR intelligence OR comprehension).mp

Embase

**Supplement or IV iron**	**Population**	**Cognitive outcomes**
**(exp diet supplementation OR fortified food.sh OR exp trace element OR formula.mp OR fortified milk.mp) AND exp iron**<br><br>OR ((iron* OR ferrous* OR ferric*) adj4 (supplement* OR compound* OR therap* OR micronutrient* OR powder OR sprinkles* OR syrup OR drop* OR tablet*OR treatment* OR formula*)).mp<br><br>OR ((IV OR intravenous OR injection OR transfusion) adj4 (Iron dextran OR Iron sucrose OR Sodium ferric gluconate OR Ferumoxytol OR Ferric carboxymaltose OR FCM OR Iron isomaltoside OR ferric derisomaltose OR Iron polymaltose OR Iron saccharate)).mp	exp pregnancy OR<br>exp reproduction OR<br>exp pregnant woman OR<br>exp fetus OR<br>exp newborn OR<br>exp prematurity OR<br>exp toddler OR<br>exp preschool child OR<br>exp progeny<br><br>OR (pregnan* OR utero OR maternal OR antenatal OR gestat* OR f?etal OR f?etus OR toddler* OR neonat* OR perinat* OR newborn* OR early childhood OR offspring OR infan* OR baby OR babies).mp	exp brain development OR<br>exp nervous system development OR<br>exp brain maturation OR<br>exp motor development OR<br>exp psychomotor development OR<br>exp mental development OR<br>speech.sh OR exp language ability OR exp oral communication OR exp "speech and language" OR<br>exp speech development OR<br>exp "speech and language assessment" OR intelligence quotient.sh OR<br>intelligence test.sh OR exp cognition OR exp nerve cell differentiation OR Comprehension.sh OR spatial memory.sh OR exp spatial orientation OR exp spatial behaviour OR exp "bayley scales of infant development" OR exp mental development assessment OR exp memory OR exp attention OR exp visual acuity<br><br>OR (neur* OR cogni* OR behavi* OR emoti* OR socioemot* OR socio-emot* OR language* OR linguistic* OR spatial learn* OR spatial navigation* OR spatial processing* OR motor* OR learn* OR mental* OR Bayley Scales* OR child development OR psychomotor OR speech OR brain OR intelligence OR comprehension).mp

Cochrane Library

**Supplement or IV iron**	**Population**	**Cognitive outcomes**
([mh iron] AND [mh “food, fortified”])<br><br>OR ((iron OR ferrous* OR ferric* OR micronutrient*) NEAR/3 (supplement* OR compound* OR therapy* OR powder* OR sprinkles* OR syrup* OR drops* OR tablet* OR treatment* OR formula*)):ti,ab,kw<br><br>OR ((IV OR intravenous OR injection OR transfusion) NEAR/3 ("Iron dextran" OR "Iron sucrose" OR "Sodium ferric gluconate" OR Ferumoxytol OR "Ferric carboxymaltose" OR FCM OR "Iron isomaltoside" OR "ferric derisomaltose" OR "Iron polymaltose" OR "Iron saccharate")):ti,ab,kw	[mh pregnancy] OR<br>[mh "child, preschool"] OR<br>[mh infant] OR<br>[mh infant, premature]<br><br>OR (pregnan* OR utero OR maternal OR antenatal OR gestat* OR f?etal OR f?etus OR toddler* OR neonat* OR perinat* OR newborn* OR early childhood OR offspring OR infan* OR baby OR babies):ti,ab,kw	[mh brain] OR<br>[mh "child development"] OR<br>[mh "psychomotor performance"] OR<br>[mh speech] OR<br>[mh "intelligence tests"] OR<br>[mh comprehension] OR<br>[mh cognition] OR<br>[mh intelligence] OR<br>[mh memory] OR<br>[mh attention] OR<br>[mh "visual acuity"] OR<br>[mh “Mental processes”] OR<br>[mh “Language development”] OR<br>[mh “Infant behaviour”] OR<br>[mh “Adolescent Development”]<br><br>OR (neur* OR cogni* OR behavi* OR emoti* OR socioemot* OR socio-emot* OR language* OR linguistic* OR spatial learn* OR spatial navigation* OR spatial processing* OR motor* OR learn* OR mental* OR "Bayley Scales*" OR "child development" OR psychomotor OR speech OR brain OR “intelligence quotient” OR behaviour OR “mental development index” OR comprehension OR “neurological function” OR “mental health” OR "neuro-developmental index" OR neuro-developmental outcomes):ti,ab,kw

CINAHL

**Supplement or IV iron**	**Population**	**Cognitive outcomes**
iron or ferrous or ferric or "iron dextran" OR "Iron sucrose" OR "Sodium ferric gluconate" OR "Ferumoxytol" OR "Ferric carboxymaltose" OR FCM OR "Iron isomaltoside" OR "ferric derisomaltose" OR "Iron polymaltose" OR "Iron saccharate"<br><br>AND (supplement* or micronutrient or "multiple micronutrient" or formula or "fortified milk" or fortified or intravenous or "i.v." or iv or injection)	pregnan* or prenatal or antenatal or perinatal or "during pregnancy" or "in utero" or gest* or trimester or infant or newborn or neonate or baby or "premature infant" or offspring or toddler* or preschool* or "early childhood"	"brain development" or "cognitive development" or neurodevelopment or "neural development" OR "motor skills" or "motor development" or "motor ability" or motoric or "fine motor" or "gross motor" or "psychomotor performance" OR "mental development" or "social development" or "cognitive development" or "moral development" OR intelligence or "intelligence quotient" or "iq" or "cognitive ability" or "cognitive development" OR "language development" or "language acquisition" or "language learning" or "speech development" OR "spatial ability" or "spatial memory" or "spatial reasoning" or "spatial navigation" or "spatial orientation" OR learning or memory or recall or cognition or "school performance" or "school achievement" or "school outcomes" or "academic achievement" OR focus or concentrat* or attent* OR behavi* or OR "mental health" or "mental illness" or "mental disorder" or "psychiatric illness" OR "developmental assessment" or "bayley scales scales of infant and toddler development"
