# Supplementary material for: The Effects of Prenatal Iron Supplementation on Offspring Neurodevelopment in Upper Middle- or High-Income Countries: A Systematic Review
Source: Nutrients. 2024 Jul 31;16(15):2499. doi: 10.3390/nu16152499 (PMC11314290; doi:10.3390/nu16152499)
Supplement: Supplementary file 1 [file nutrients-16-02499-s001.zip › Table S3 Excluded Studies.pdf]

Table S3. Excluded Studies

Citation	Reason Excluded
Angulo-Barroso 2016 [1]	LMIC[a]
Aukett 1986 [2]	Ineligible population (infants)
Baumgartner 2012 [3]	Ineligible population (animal)
Berglund 2011 [4]	Ineligible population (infants)
Berglund 2013 [5]	Ineligible population (infants)
Berglund 2018 [6]	Ineligible population (infants)
Black 2004 [7]	LMIC[a]
Bora 2021 [8]	LMIC[a]
Bouhouch 2016 [9]	LMIC[a]
Doom 202 [10]	LMIC[a]
East 2022 [11]	LMIC[a]
East 2023 [12]	LMIC[a]
Friel 2001 [13]	Ineligible population (infants)
Friel 2003 [14]	Ineligible population (infants)
Gahagan 2017 [15]	Abstract only
Gahagan 2019 [16]	LMIC[a]
Griffin 1999 [17]	Ineligible population (infants)
Harahap 2000 [18]	LMIC[a]
Heywood 1989 [19]	LMIC[a]
Honig 1978 [20]	Ineligible population (infants)
Idjradinata 1993 [21]	LMIC[a]
Iglesisas-Vazquez 2019 [22]	Ineligible population (infants)
Jahari 2000 [23]	LMIC[a]
Lind 2004 [24]	LMIC[a]
Lozoff 1982 [25]	LMIC[a]
Lozoff 1987 [26]	LMIC[a]
Lozoff 1996 [27]	LMIC[a]
Lozoff 2003 [28]	LMIC[a]
Lozoff 2012 [29]	LMIC[a]
Lozoff 2014 [30]	LMIC[a]
Luciano, 2022 [31]	Ineligible population (infants)
Miller 2013 [32]	Ineligible population (infants)
Moffatt 1994 [33]	Ineligible population (infants)
Morley 1999 [34]	Ineligible population (infants)
Nguyen, 2017 [35]	LMIC[a]
Nguyen 2021 [36]	LMIC[a]
Oski 1978 [37]	Ineligible population (infants)
Parkin 2021[38]	Ineligible population (toddlers)
Pasricha 2021[39]	LMIC[a]
Pongcharoen 2011[40]	LMIC[a]
Rico 2006 [41]	Ineligible population (school aged children)

Soewondo 1989 [42]	LMIC[a]
Ssemata 2020 [43]	LMIC[a]
Steinmacher 2007 [44]	Ineligible population (infants)
Stoltzfus 2001 [45]	LMIC[a]
Walter 1989 [46]	LMIC[a]
Walter 1983 [47]	LMIC[a]
Warthon-Medina, 2015 [48]	LMIC[a]
Williams 1999 [49]	Ineligible population (infants)
Yalcin 2000 [50]	Ineligible population (infants)
Zhu 2018 [51]	LMIC[a]
Zhu 2020 [52]	LMIC[a]
Zhu 2023 [53]	LMIC[a]

[a] Trial was conducted in a low- or middle-income country. This was the first exclusion criteria applied; more than one criterion may apply e.g., LMIC and ineligible intervention.

References

1. Angulo-Barroso RM, Li M, Santos DCC, Bian Y, Sturza J, Jiang Y, et al. Iron supplementation in pregnancy or infancy and motor development: a randomized controlled trial. Pediatrics 2016;137(4).
2. Aukett MA, Parks YA, Scott PH, Wharton BA. Treatment with iron increases weight gain and psychomotor development. Arch Dis Child 1986;61(9):849-57.
3. Baumgartner J, Smuts CM, Malan L, Kvalsvig J, van Stuijvenberg ME, Hurrell RF, et al. Effects of iron and n-3 fatty acid supplementation, alone and in combination, on cognition in school children: a randomized, double-blind, placebo-controlled intervention in South Africa. Am J Clin Nutr 2012;96(6):1327-38.
4. Berglund SK, Westrup B, Haraldsson E, Engstrom B, Domellof M. Effects of iron supplementation on auditory brainstem response in marginally LBW infants. Pediatr. Res 2011;70(6):601-6.
5. Berglund SK, Westrup B, Hagglof B, Hernell O, Domellof M. Effects of iron supplementation of LBW infants on cognition and behavior at 3 years. Pediatrics 2013;131(1):47-55.
6. Berglund SK, Chmielewska A, Starnberg J, Westrup B, Hagglof B, Norman M, et al. Effects of iron supplementation of low-birth-weight infants on cognition and behavior at 7 years: a randomized controlled trial. Pediatr. Res 2018;83(1-1):111-8.
7. Black MM, Baqui AH, Zaman K, Ake Persson L, El Arifeen S, Le K, et al. Iron and zinc supplementation promote motor development and exploratory behavior among Bangladeshi infants. Am J Clin Nutr 2004;80(4):903-10.
8. Bora R, Ramasamy S, Brown B, Wolfson J, Rao R. Effect of iron supplementation from neonatal period on the iron status of6-month-old infants at-risk for early iron deficiency: a randomized interventional trial. J Matern Fetal Neonatal Med 2021;34(9):1421-9.
9. Bouhouch RR, El-Fadeli S, Andersson M, Aboussad A, Chabaa L, Zeder C, et al. Effects of wheat-flour biscuits fortified with iron and EDTA, alone and in combination, on blood lead concentration, iron status, and cognition in children: a double-blind randomized controlled trial. Am J Clin Nutr 2016;104(5):1318-26.
10. Doom JR, Gahagan S, Caballero G, Encina P, Lozoff B. Infant iron deficiency, iron supplementation, and psychosocial stress as predictors of neurocognitive development in Chilean adolescents. Nutr Neurosci 2021;24(7):520-9.
11. East P, Doom J, Blanco E, Burrows R, Lozoff B, Gahagan S. Young adult outcomes associated with lower cognitive functioning in childhood related to iron-fortified formula in infancy. Nutr Neurosci 2022;25(4):709-18.
12. East PL, Reid B, Blanco E, Burrows R, Lozoff B, Gahagan S. Iron supplementation given to nonanemic infants: neurocognitive functioning at 16 years. Nutr Neurosci 2023;26(1):40-9.
13. Friel JK, Andrews WL, Aziz K, Kwa PG, Lepage G, L'Abbe MR. A randomized trial of two levels of iron supplementation and developmental outcome in low birth weight infants. J Pediatr 2001;139(2):254-60.
14. Friel JK, Aziz K, Andrews WL, Harding SV, Courage ML, Adams RJ. A double-masked, randomized control trial of iron supplementation in early infancy in healthy term breast-fed infants. J Pediatr 2003;143(5):582-6.
15. Gahagan S, Delker E, Castillo M, Lozoff B. Iron-fortified vs low-iron infant formula: cognitive outcomes at 10 and 16 years. [https://onlinelibrary.wiley.com/doi/pdf/10.1002/ajh.24812]. Accessed 7 March 2024.

16. Gahagan S, Delker E, Blanco E, Burrows R, Lozoff B. Randomized controlled trial of iron-fortified versus low-iron infant formula: developmental outcomes at 16 Years. J Pediatr 2019;212:124-30.e1.
17. Griffin IJ, Cooke RJ, Reid MM, McCormick KP, Smith JS. Iron nutritional status in preterm infants fed formulas fortified with iron. Arch Dis Child Fetal Neonatal Ed 1999;81(1):F45-9.
18. Harahap H, Jahari AB, Husaini MA, Saco-Pollitt C, Pollitt E. Effects of an energy and micronutrient supplement on iron deficiency anemia, physical activity and motor and mental development in undernourished children in Indonesia. Eur J clin Nutr 2000;54 Suppl 2:S114-9.
19. Heywood A, Oppenheimer S, Heywood P, Jolley D. Behavioral effects of iron supplementation in infants in Madang, Papua New Guinea. Am J Clin Nutr 1989;50(3 Suppl):630-40.
20. Honig AS, Oski FA. Developmental scores of iron deficeint infants and the effects of therapy. Infant Behav Dev 1978;1(1):168-76.
21. Idjradinata P, Pollitt E. Reversal of developmental delays in iron-deficient anaemic infants treated with iron. Lancet 1993;341(8836):1-4.
22. Iglesias Vazquez L, Canals J, Voltas N, Jardi C, Hernandez C, Bedmar C, et al. Does the fortified milk with high iron dose improve the neurodevelopment of healthy infants? Randomized controlled trial. BMC Pediatr 2019;19(1):315.
23. Jahari AB, Saco-Pollitt C, Husaini MA, Pollitt E. Effects of an energy and micronutrient supplement on motor development and motor activity in undernourished children in Indonesia. Eur J Clin Nutr 2000;54 Suppl 2:S60-8.
24. Lind T, Lonnerdal B, Stenlund H, Gamayanti IL, Ismail D, Seswandhana R, et al. A community-based randomized controlled trial of iron and zinc supplementation in Indonesian infants: effects on growth and development. Am J Clin Nutr 2004;80(3):729-36.
25. Lozoff B, Brittenham GM, Viteri FE, Wolf AW, Urrutia JJ. The effects of short-term oral iron therapy on developmental deficits in iron-deficient anemic infants. J Pediatr 1982;100(3):351-7.
26. Lozoff B, Brittenham GM, Wolf AW, McClish DK, Kuhnert PM, Jimenez E, et al. Iron deficiency anemia and iron therapy effects on infant developmental test performance. Pediatrics 1987;79(6):981-95.
27. Lozoff B, Wolf AW, Jimenez E. Iron-deficiency anemia and infant development: effects of extended oral iron therapy. J Pediatr 1996;129(3):382-9.
28. Lozoff B, De Andraca I, Castillo M, Smith JB, Walter T, Pino P. Behavioral and developmental effects of preventing iron-deficiency anemia in healthy full-term infants. Pediatrics 2003;112(4):846-54.
29. Lozoff B, Castillo M, Clark KM, Smith JB. Iron-fortified vs low-iron infant formula: developmental outcome at 10 years. Arch Pediatr Adolesc Med 2012;166(3):208-15.
30. Lozoff B, Castillo M, Clark KM, Smith JB, Sturza J. Iron supplementation in infancy contributes to more adaptive behavior at 10 years of age. J Nutr 2014;144(6):838-45.
31. Luciano R, Romeo DM, Mancini G, Sivo S, Dolci C, Velli C, et al. Neurological development and iron supplementation in healthy late-preterm neonates: a randomized double-blind controlled trial. Eur J Pediatr 2022;181(1):295-302.
32. Miller SM. Iron supplementation in premature infants using the zinc protoporphyrin to heme ratio: short- and long-term outcomes. J Perinatol 2013;33(9):712-6.
33. Moffatt ME, Longstaffe S, Besant J, Dureski C. Prevention of iron deficiency and psychomotor decline in high-risk infants through use of iron-fortified infant formula: a randomized clinical trial. J Pedatr 1994;125(4):527-34.

34. Morley R, Abbott R, Fairweather-Tait S, MacFadyen U, Stephenson T, Lucas A. Iron fortified follow on formula from 9 to 18 months improves iron status but not development or growth: a randomised trial. Arch Dis Child 1999;81(3):247-52.
35. Nguyen PH, Gonzalez-Casanova I, Young MF, Truong TV, Hoang H, Nguyen H, et al. Preconception micronutrient supplementation with iron and folic acid compared with folic acid alone affects linear growth and fine motor development at 2 years of age: a randomized controlled trial in Vietnam. J Nutr 2017;147(8):1593-601.
36. Nguyen PH, Young MF, Tran LM, Khuong LQ, Duong TH, Nguyen HC, et al. Preconception micronutrient supplementation positively affects child intellectual functioning at 6 y of age: a randomized controlled trial in Vietnam. Am J Clin Nutr 2021;113(5):1199-208.
37. Oski FA, Honig AS. The effects of therapy on the developmental scores of iron-deficient infants. J Pediatr 1978;92(1):21-5.
38. Parkin PC, Borkhoff CM, Macarthur C, Abdullah K, Birken CS, Fehlings D, et al. Randomized trial of oral iron and diet advice versus diet advice alone in young children with nonanemic iron deficiency. J Pediatr 2021;233:233-40.e1.
39. Pasricha S-R, Hasan MI, Braat S, Larson LM, Tipu SMM-U, Hossain SJ, et al. Benefits and risks of iron interventions in infants in rural Bangladesh. N Engl J Med 2021;385(11):982-95.
40. Pongcharoen T, DiGirolamo AM, Ramakrishnan U, Winichagoon P, Flores R, Martorell R. Long-term effects of iron and zinc supplementation during infancy on cognitive function at 9 y of age in northeast Thai children: a follow-up study. Am J Clin Nutr 2011;93(3):636-43.
41. Rico JA, Kordas K, Lopez P, Rosado JL, Vargas GG, Ronquillo D, et al. Efficacy of iron and/or zinc supplementation on cognitive performance of lead-exposed Mexican schoolchildren: a randomized, placebo-controlled trial. Pediatrics 2006;117(3):e518-27.
42. Soewondo S, Husaini M, Pollitt E. Effects of iron deficiency on attention and learning processes in preschool children: Bandung, Indonesia. Am J Clin Nutr 1989;50(3 Suppl):667-4.
43. Ssemata AS, Hickson M, Ssenkusu JM, Cusick SE, Nakasujja N, Opoka RO, et al. Delayed iron does not alter cognition or behavior among children with severe malaria and iron deficiency. Pediatr Res 2020;88(3):429-37.
44. Steinmacher J, Pohlandt F, Bode H, Sander S, Kron M, Franz AR. Randomized trial of early versus late enteral iron supplementation in infants with a birth weight of less than 1301 grams: neurocognitive development at 5.3 years' corrected age. Pediatrics 2007;120(3):538-46.
45. Stoltzfus RJ, Kvalsvig JD, Chwaya HM, Montresor A, Albonico M, Tielsch JM, et al. Effects of iron supplementation and anthelmintic treatment on motor and language development of preschool children in Zanzibar: double blind, placebo controlled study. BMJ 2001;323(7326):1389-93.
46. Walter T, De Andraca I, Chadud P, Perales CG. Iron deficiency anemia: adverse effects on infant psychomotor development. Pediatrics 1989;84(1):7-17.
47. Walter T, Kovalskys J, Stekel A. Effect of mild iron deficiency on infant mental development scores. J Pediatr 1983;102(4):519-22.
48. Warthon-Medina M, Qualter P, Zavaleta N, Dillon S, Lazarte F, Lowe NM. The long term impact of micronutrient supplementation during infancy on cognition and executive function performance in pre-school children. Nutrients 2015;7(8):6606-27.

49. Williams J, Wolff A, Daly A, MacDonald A, Aukett A, Booth IW. Iron supplemented formula milk related to reduction in psychomotor decline in infants from inner city areas: randomised study. BMJ 1999;318(7185):693-7.
50. Yalcin SS, Yurdakok K, Acikgoz D, Ozmert E. Short-term developmental outcome of iron prophylaxis in infants. Pediatr Int 2000;42(6):625-30.
51. Zhu Z, Cheng Y, Zeng L, Elhoumed M, He G, Li W, et al. Association of antenatal micronutrient supplementation with adolescent intellectual development in rural western China: 14-Year follow-up from a randomized clinical trial. JAMA Pediatr 2018;172(9):832-41.
52. Zhu Z, Cheng Y, Qi Q, Lu Y, Ma S, Li S, et al. Association of infant and young child feeding practices with cognitive development at 10-12 years: a birth cohort in rural Western China. Br J Nutr 2020;123(7):768-79.
53. Zhu Z, Zhu Y, Wang L, Qi Q, Huang L, Andegiorgish AK, et al. Effects of antenatal micronutrient supplementation regimens on adolescent emotional and behavioral problems: A 14-year follow-up of a double-blind, cluster-randomized controlled trial. Clin Nutr 2023;42(2):129-35.
