# Supplementary material for: The Effects of Prenatal Iron Supplementation on Offspring Neurodevelopment in Upper Middle- or High-Income Countries: A Systematic Review
Source: Nutrients. 2024 Jul 31;16(15):2499. doi: 10.3390/nu16152499 (PMC11314290; doi:10.3390/nu16152499)
Supplement: Supplementary file 1 [file nutrients-16-02499-s001.zip › Table S4 Risk of Bias Rationale.pdf]

Supplementary Table S4. Risk of bias rationale for judgment

Author, year		Selection bias (random sequence generation)	Selection bias (allocation concealment)	Performance bias (blinding of participants and personnel)	Detection bias (blinding of outcome assessment)	Attrition bias (incomplete outcome data)	Reporting bias (selective outcome reporting)	Other bias
**Prevention of IDA**								
AMBIT RCT	Zhou 2006 [1]	Low risk<br><br>Computer-generated randomization schedule consultant.	Low risk<br><br>Opaque bottles marked with a sequential, numerical code	Low risk<br><br>Families and research staff blinded to group assignment	Low risk<br><br>Families and research staff blinded to group assignment until analyses complete	High risk<br><br>High attrition at four years<br><br>20 mg iron: 30% (66/219)<br>Placebo: 30% (65/214))	Unclear risk<br><br>Insufficient information re outcome reporting. Early study, no trial registration or protocol publication.	Low risk<br><br>Intent-to-treat analysis
	Parsons 2008 [2]			High risk<br><br>Parents were unblinded after four-year follow-up.	High risk<br><br>Parents were unblinded and completed assessments on their children.	High risk<br><br>High attrition at six to eight years<br><br>20mg iron: 38% (84/219)<br>Placebo: 38% (82/214)		

ECLIPSES RCT	Iglesias-Vazquez 2022 [3]	Low risk<br><br>Randomization performed using centralised computer software.	Low risk<br><br>Randomisation procedure is automated, masked, and independent for each stratum.	Low risk<br><br>Triple blinded (participant, health care professional and statistician).	Low risk<br><br>Health professional and statistician also blinded.	High attrition at 40 days<br><br>*Baseline Hb 110-130g/L*<br><br>80 mg iron: 40% (107/268)<br>40 mg iron: 36% (94/261))<br><br>*Baseline Hb > 130 g/L*<br><br>40 mg iron: 30% (39/132)<br>20 mg iron :37% (48/130)	High risk<br><br>Trial registration and the published trial protocol indicate both intent-to-treat and per-protocol analysis, but only latter published.	High risk<br><br>All results are based on per-protocol analysis. Intent-to-treat analyses requested but unavailable.
	Iglesias-Vazquez 2023 [4]					*Baseline Hb 110-130g/L*<br><br>80 mg iron: 64% (172/268)<br>40 mg iron: 66% (171/261)<br><br>*Baseline Hb > 130 g/L*<br><br>40 mg iron: 56% (74/132)<br>20 mg iron: 61% (79/130)	Unclear risk<br><br>Methods of follow up at four years not outlined in the trial registration or published protocol.	High risk<br><br>Per-protocol results published; however, authors provided intent-treat-analyses upon request.

Treatment of ID							
IV Iron RCT	Froessler 2022 [5]	Low risk<br><br>Randomisation by trial pharmacist using an online randomisation sequence generator	Low risk<br><br>Sequence generation with opaque envelopes	Low risk<br><br>Participants, clinicians, researchers, and statistician masked to allocation until analyses complete	Low risk<br><br>Researchers and statistician blinded until analyses complete	High risk<br><br>High attrition at 12 months for neurodevelopmental outcomes ~50%	Unclear risk<br><br>Neurodevelopmental assessments were not included in the trial registration and trial protocol was not published.	Low risk<br><br>Intent-to-treat analysis

Abbreviations: Hb: haemoglobin; ID: iron deficiency; IDA: iron deficiency anaemia; IV: intravenous; RCT: randomised controlled trial.

References

1. Zhou, S.J.; Gibson, R.A.; Crowther, C.A.; Baghurst, P.; Makrides, M. Effect of iron supplementation during pregnancy on the intelligence quotient and behavior of children at 4 y of age: long-term follow-up of a randomized controlled trial. *Am J Clin Nutr* **2006**, *83*, 1112-1117.
2. Parsons, A.G.; Zhou, S.J.; Spurrier, N.J.; Makrides, M. Effect of iron supplementation during pregnancy on the behaviour of children at early school age: long-term follow-up of a randomised controlled trial. *Br J Nutr* **2008**, *99*, 1133-1139.
3. Iglesias-Vazquez, L.; Hernandez-Martinez, C.; Voltas, N.; Canals, J.; Coronel, P.; Gimeno, M.; Arija, V. Adapting prenatal iron supplementation to maternal needs results in optimal child neurodevelopment: a follow-up of the ECLIPSES Study. *BMC Pregnancy Childbirth* **2022**, *22*, 710, doi:https://dx.doi.org/10.1186/s12884-022-05033-y.
4. Iglesias-Vazquez, L.; Voltas, N.; Hernandez-Martinez, C.; Canals, J.; Coronel, P.; Gimeno, M.; Basora, J.; Arija, V. Importance of Maternal Iron Status on the Improvement of Cognitive Function in Children After Prenatal Iron Supplementation. *Am J Prev Med* **2023**, https://dx.doi.org/10.1016/j.amepre.2023.02.006, doi:https://dx.doi.org/10.1016/j.amepre.2023.02.006.
5. Froessler, B.; Schubert, K.O.; Palm, P.; Church, R.; Aboustate, N.; Kelly, T.L.; Dekker, G.A.; Hodyl, N.A. Testing equivalence of two doses of intravenous iron to treat iron deficiency in pregnancy: A randomised controlled trial. *BJOG* **2023**, *130*, 15-23, doi:10.1111/1471-0528.17288.
