# Supplementary material for: The Effects of Prenatal Iron Supplementation on Offspring Neurodevelopment in Upper Middle- or High-Income Countries: A Systematic Review
Source: Nutrients. 2024 Jul 31;16(15):2499. doi: 10.3390/nu16152499 (PMC11314290; doi:10.3390/nu16152499)
Supplement: Supplementary file 1 [file nutrients-16-02499-s001.zip › Table S5 Secondary Outcomes.pdf]

## Table S5. Summary of results: secondary outcomes

| Secondary Outcomes | Number of Participants (RCTs) | Effect | |
|---|---|---|---|
| | | Risk Ratio (95% CI) | Mean Difference (95% CI) |
| **Prevention of IDA: language** | | | |
| Language development (Bayley-III at 40 days) | | | |
| Baseline Hb 110-130 g/L | | | |
| 80 mg versus 40 mg oral iron | 328 (1 RCT) [1] | -- | 0.52 (-1.31, 2.35) |
| Baseline Hb > 130 g/L | | | |
| 40 mg versus 20 mg oral iron | 175 (1 RCT) [1] | -- | 2.30 (-0.01, 4.61) |
| Language development score <85 (Bayley-III at 40 days) | | | |
| Baseline Hb 110-130 g/L | | | |
| 80 mg versus 40 mg oral iron | 328 (1 RCT) [1] | 0.79 (0.40, 1.58) | -- |
| Baseline Hb > 130 g/L | | | |
| 40 mg versus 20 mg oral iron | 175 (1 RCT) [1] | 0.50 (0.15, 1.66) | -- |
| Expressive language (Bayley-III at 40 days) | | | |
| Baseline Hb 110-130 g/L | | | |
| 80 mg versus 40 mg oral iron | 328 (1 RCT) [1] | -- | -0.12 (-0.45, 0.21) |
| Baseline Hb > 130 g/L | | | |
| 40 mg versus 20 mg oral iron | 175 (1 RCT) [1] | -- | 0.21 (-0.26, 0.68) |
| Expressive language score < 7 (Bayley-III at 40 days) | | | |
| Baseline Hb 110-130 g/L | | | |
| 80 mg versus 40 mg oral iron | 328 (1 RCT) [1] | 0.73 (0.38, 1.39) | -- |
| Baseline Hb > 130 g/L | | | |
| 40 mg versus 20 mg oral iron | 175 (1 RCT) [1] | 1.67 (0.77, 3.62) | -- |
| Receptive language (Bayley-III at 40 days) | | | |
| Baseline Hb 110-130 g/L | | | |
| 80 mg versus 40 mg oral iron | 328 (1 RCT) [1] | -- | 0.30 (-0.16, 0.76) |
| Baseline Hb > 130 g/L | | | |
| 40 mg versus 20 mg oral iron | 175 (1 RCT) [1] | -- | 0.55 (-0.06, 1.16) |

| | | | |
|---|---|---|---|
| Receptive language score < 7 (Bayley-III at 40 days) | | | |
| Baseline Hb 110-130 g/L | | | |
| 80 mg versus 40 mg oral iron | 328 (1 RCT) [1] | 1.04 (0.37, 2.89) | -- |
| Baseline Hb > 130 g/L | | | |
| 40 mg versus 20 mg oral iron | 175 (1 RCT) [1] | 1.76 (0.33, 9.38) | -- |
| **Treatment of ID: language** | | | |
| Communication (ASQ at 12 months) | | | |
| Baseline Hb 110-130 g/L | | | |
| 1000 mg versus 500 mg IV FCM | 130 (1 RCT) [2] | 2.00 (-1.64, 5.64) | -- |
| **Prevention of IDA: language** | | | |
| Verbal reasoning (SBIS at four years) | | | |
| Baseline Hb > 110 g/L | | | |
| 20 mg oral iron versus placebo | 302 (1 RCT) [3] | -- | 0.00 (-2.60, 2.60) |
| Verbal comprehension index (WPPSI-IV at four years) | | | |
| Baseline Hb 110-130 g/L | | | |
| 80 mg versus 40 mg oral iron | 182 (1 RCT) [4] | -- | -1.10 (-5.01, 2.81) |
| Baseline Hb > 130 g/L | | | |
| 40 mg versus 20 mg oral iron | 106 (1 RCT) [4] | -- | 1.41 (-3.40, 6.22) |
| Vocabulary acquisition index (WPPSI-IV at four years) | | | |
| Baseline Hb 110-130 g/L | | | |
| 80 mg versus 40 mg oral iron | 182 (1 RCT) [4] | -- | -2.39 (-6.49, 1.71) |
| Baseline Hb > 130 g/L | | | |
| 40 mg versus 20 mg oral iron | 106 (1 RCT) [4] | -- | 0.82 (-4.30, 5.94) |
| Non-verbal index (WPPSI-IV at four years) | | | |
| Baseline Hb 110-130 g/L | | | |
| 80 mg versus 40 mg oral iron | 182 (1 RCT) [4] | -- | 1.91 (-1.89, 5.71) |
| Baseline Hb > 130 g/L | | | |
| 40 mg versus 20 mg oral iron | 106 (1 RCT) [4] | -- | 0.19 (-3.92, 4.30) |
| Verbal fluency (WPPSI-IV at four years) | | | |
| Baseline Hb 110-130 g/L | | | |
| 80 mg versus 40 mg oral iron | 182 (1 RCT) [4] | -- | -0.66 (-1.49, 0.17) |

| | | | |
|---|---|---|---|
| Baseline Hb > 130 g/L | | | |
| 40 mg versus 20 mg oral iron | 106 (1 RCT) [4] | -- | -0.44 (-1.45, 0.57) |
| **Prevention of IDA: motor development** | | | |
| Motor development (Bayley-III at 40 days) | | | |
| Baseline Hb 110-130 g/L | | | |
| 80 mg versus 40 mg oral iron | 328 (1 RCT) [1] | -- | -0.56 (-3.07, 1.95) |
| Baseline Hb > 130 g/L | | | |
| 40 mg versus 20 mg oral iron | 175 (1 RCT) [1] | -- | 0.92 (-2.25, 4.09) |
| Motor development score < 85 (Bayley-III at 40 days) | | | |
| Baseline Hb 110-130 g/L | | | |
| 80 mg versus 40 mg oral iron | 328 (1 RCT) [1] | 1.30 (0.35, 4.74) | -- |
| Baseline Hb > 130 g/L | | | |
| 40 mg versus 20 mg oral iron | 175 (1 RCT) [1] | 1.76 (0.33, 9.38) | -- |
| Gross motor (Bayley-III at 40 days) | | | |
| Baseline Hb 110-130 g/L | | | |
| 80 mg versus 40 mg oral iron | 328 (1 RCT) [1] | -- | 0.05 (-0.16, 0.27) |
| Baseline Hb > 130 g/L | | | |
| 40 mg versus 20 mg oral iron | 175 (1 RCT) [1] | -- | 0.07 (-0.22, 0.37) |
| Gross motor development score < 7 (Bayley-III at 40 days) | | | |
| Baseline Hb 110-130 g/L | | | |
| 80 mg versus 40 mg oral iron | 328 (1 RCT) [1] | 0.35 (0.01, 8.42) | -- |
| Baseline Hb > 130 g/L | | | |
| 40 mg versus 20 mg oral iron | 175 (1 RCT) [1] | Not estimable | |
| Fine motor development (Bayley-III at 40 days) | | | |
| Baseline Hb 110-130 g/L | | | |
| 80 mg versus 40 mg oral iron | 328 (1 RCT) [1] | -- | -0.04 (-0.25, 0.18) |
| Baseline Hb > 130 g/L | | | |
| 40 mg versus 20 mg oral iron | 175 (1 RCT) [1] | -- | 0.02 (-0.28, 0.32) |
| Fine motor development score < 7 (Bayley-III at 40 days) | | | |
| Baseline Hb 110-130 g/L | | | |

| | | | |
|---|---|---|---|
| 80 mg versus 40 mg oral iron | 328 (1 RCT) [1] | 1.04 (0.07, 16.44) | -- |
| Baseline Hb > 130 g/L | | | |
| 40 mg versus 20 mg oral iron | 175 (1 RCT) [1] | 1.76 (0.16, 19.09) | -- |
| **Treatment of ID: motor development** | | | |
| Gross motor (ASQ at 12 months) | | | |
| Baseline Hb 110-130 g/L | | | |
| 1000 mg versus 500 mg IV FCM | 130 (1 RCT) [2] | -- | 0.06 (-0.29, 0.41) |
| Fine motor (ASQ AT 12 MONTHS) | | | |
| Baseline Hb 110-130 g/L | | | |
| 1000 mg versus 500 mg IV FCM | 130 (1 RCT) [2] | -- | 0.10 (-0.25, 0.45) |
| **Treatment of ID: emotional behavioural functioning** | | | |
| Personal social development (ASQ at 12 months) | | | |
| Baseline Hb 110-130 g/L | | | |
| 1000 mg versus 500 mg IV FCM | 134 (1 RCT) [2] | -- | 0.08 (-0.27, 0.42) |
| **Prevention of IDA: emotional behavioural functioning** | | | |
| Emotion (parent-rated SDQ at four years) | | | |
| Baseline Hb > 110 g/L | | | |
| 20 mg oral iron versus placebo | 302 (1 RCT) [3] | -- | 0.10 (-0.31, 0.51) |
| Conduct (parent-rated SDQ at four years) | | | |
| Baseline Hb > 110 g/L | | | |
| 20 mg oral iron versus placebo | 302 (1 RCT) [3] | -- | 0.10 (-0.34, 0.54) |
| Hyperactivity (parent-rated SDQ at four years) | | | |
| Baseline Hb > 110 g/L | | | |
| 20 mg oral iron versus placebo | 302 (1 RCT) [3] | -- | 0.00 (-0.50, 0.50) |
| Peer relations (parent-rated SDQ at four years) | | | |
| Baseline Hb > 110 g/L | | | |
| 20 mg oral iron versus placebo | 302 (1 RCT) [3] | -- | 0.40 (0.04, 0.76) |
| Total behavioural difficulties score (parent-rated SDQ at four years) | | | |
| Baseline Hb > 110 g/L | | | |
| 20 mg oral iron versus placebo | 302 (1 RCT) [3] | -- | 0.40 (-0.79, 1.59) |

| | | | |
|---|---|---|---|
| Total difficulties score ≥ 17 (parent-rated SDQ at four years) | | | |
| Baseline Hb > 110 g/L | | | |
| 20 mg oral iron versus placebo | 302 (1 RCT) [3] | 1.95 (1.01, 3.75) | -- |
| Emotion recognition (NEPSY-II at four years) | | | |
| Baseline Hb 110-130 g/L | | | |
| 80 mg versus 40 mg oral iron | 182 (1 RCT) [4] | -- | -0.62 (-1.35, 0.11) |
| Baseline Hb > 130 g/L | | | |
| 40 mg versus 20 mg oral iron | 106 (1 RCT) [4] | -- | 0.04 (-0.93, 1.01) |
| **Prevention of IDA: emotional behavioural functioning** | | | |
| Emotion (parent-rated SDQ at six to eight years) | | | |
| Baseline Hb > 110 g/L | | | |
| 20 mg oral iron versus placebo | 264 (1 RCT) [5] | -- | -0.30 (-0.81, 0.21) |
| Emotion score ≥ 5 (parent-rated SDQ at six to eight years) | | | |
| Baseline Hb > 110 g/L | | | |
| 20 mg oral iron versus placebo | 264 (1 RCT) [5] | 1.13 (0.60, 2.11) | -- |
| Conduct (parent-rated SDQ at six to eight years) | | | |
| Baseline Hb > 110 g/L | | | |
| 20 mg oral iron versus placebo | 264 (1 RCT) [5] | -- | -0.20 (-0.65, 0.25) |
| Conduct score ≥ 4 (parent-rated SDQ at six to eight years) | | | |
| Baseline Hb > 110 g/L | | | |
| 20 mg oral iron versus placebo | 264 (1 RCT) [5] | 0.87 (0.50, 1.50) | -- |
| Hyperactivity (parent-rated SDQ at six to eight years) | | | |
| Baseline Hb > 110 g/L | | | |
| 20 mg oral iron versus placebo | 264 (1 RCT) [5] | -- | 0.00 (-0.66, 0.66) |
| Hyperactivity score ≥ 7 (parent-rated SDQ at six to eight years) | | | |
| Baseline Hb > 110 g/L | | | |
| 20 mg oral iron versus placebo | 264 (1 RCT) [5] | 1.05 (0.61, 1.78) | -- |

| | | | |
|---|---|---|---|
| Peer problems (parent-rated SDQ at six to eight years) | | | |
| Baseline Hb > 110 g/L | | | |
| 20 mg oral iron versus placebo | 264 (1 RCT) [5] | -- | 0.00 (-0.39, 0.39) |
| Peer problems score ≥ 4 (parent-rated SDQ at six to eight years) | | | |
| Baseline Hb > 110 g/L | | | |
| 20 mg oral iron versus placebo | 264 (1 RCT) [5] | 1.23 (0.62, 2.46) | -- |
| Pro-social behaviour (parent-rated SDQ at six to eight years) | | | |
| Baseline Hb > 110 g/L | | | |
| 20 mg oral iron versus placebo | 264 (1 RCT) [5] | -- | 0.10 (-0.35, 0.55) |
| Pro-social behaviour score ≤ 4 (parent-rated SDQ at six to eight years) | | | |
| Baseline Hb > 110 g/L | | | |
| 20 mg oral iron versus placebo | 264 (1 RCT) [5] | 1.17 (0.40, 3.38) | -- |
| Total behavioural difficulties score (parent-rated SDQ at six to eight years) | | | |
| Baseline Hb > 110 g/L | | | |
| 20 mg oral iron versus placebo | 264 (1 RCT) [5] | -- | -0.60 (-2.07, 0.87) |
| Total difficulties score ≥ 17 (parent-rated SDQ at six to eight years) | | | |
| Baseline Hb > 110 g/L | | | |
| 20 mg oral iron versus placebo | 264 (1 RCT) [5] | 0.88 (0.46, 1.69) | -- |
| Emotion (teacher-rated SDQ at six to eight years) | | | |
| Baseline Hb > 110 g/L | | | |
| 20 mg oral iron versus placebo | 225 (1 RCT) [5] | -- | -0.30 (-0.83, 0.23) |
| Emotion score ≥5 (teacher-rated SDQ at six to eight years) | | | |
| Baseline Hb > 110 g/L | | | |
| 20 mg oral iron versus placebo | 225 (1 RCT) [5] | 0.45 (0.14, 1.41) | -- |
| Conduct (teacher-rated SDQ at six to eight years) | | | |

| | | | |
|---|---|---|---|
| Baseline Hb > 110 g/L | | | |
| 20 mg oral iron versus placebo | 225 (1 RCT) [5] | -- | 0.10 (-0.35, 0.55) |
| Conduct score ≥ 4 (teacher-rated SDQ at six to eight years) | | | |
| Baseline Hb > 110 g/L | | | |
| 20 mg oral iron versus placebo | 225 (1 RCT) [5] | 1.14 (0.45, 2.84) | -- |
| Hyperactivity (teacher-rated SDQ at six to eight years) | | | |
| Baseline Hb > 110 g/L | | | |
| 20 mg oral iron versus placebo | 225 (1 RCT) [5] | -- | -0.10 (-0.90, 0.70) |
| Hyperactivity score ≥ 7 (teacher-rated SDQ at six to eight years) | | | |
| Baseline Hb > 110 g/L | | | |
| 20 mg oral iron versus placebo | 225 (1 RCT) [5] | 0.55 (0.29, 1.06) | -- |
| Peer problems (teacher-rated SDQ at six to eight years) | | | |
| Baseline Hb > 110 g/L | | | |
| 20 mg oral iron versus placebo | 225 (1 RCT) [5] | -- | 0.20 (-0.24, 0.64) |
| Peer problems score ≥ 4 (teacher-rated SDQ at six to eight years) | | | |
| Baseline Hb > 110 g/L | | | |
| 20 mg oral iron versus placebo | 225 (1 RCT) [5] | 3.70 (1.06, 12.91) | -- |
| Pro-social behaviour (teacher-rated SDQ at six to eight years) | | | |
| Baseline Hb > 110 g/L | | | |
| 20 mg oral iron versus placebo | 225 (1 RCT) [5] | -- | 0.20 (-0.39, 0.79) |
| Pro-social behaviour score ≤ 4 (teacher-rated SDQ at six to eight years) | | | |
| Baseline Hb > 110 g/L | | | |
| 20 mg oral iron versus placebo | 225 (1 RCT) [5] | 0.65 (0.29, 1.44) | |
| Total behavioural difficulties score (teacher-rated SDQ at six to eight years) | | | |
| Baseline Hb >110 g/L | | | |

| | | | |
|---|---|---|---|
| 20 mg oral iron versus placebo | 225 (1 RCT) [5] | -- | -0.10 (-1.73, 1.53) |
| Total difficulties score ≥ 17 (teacher-rated SDQ at six to eight years) | | | |
| Baseline Hb > 110 g/L | | | |
| 20 mg oral iron versus placebo | 225 (1 RCT) [5] | 0.78 (0.36, 1.70) | -- |
| Approach (parent-rated STS at six to eight years) | | | |
| Baseline Hb > 110 g/L | | | |
| 20 mg oral iron versus placebo | 264 (1 RCT) [5] | -- | -0.10 (-0.34, 0.14) |
| Approach score >1 SD above mean (parent-rated STS at six to eight years) | | | |
| Baseline Hb > 110 g/L | | | |
| 20 mg oral iron versus placebo | 264 (1 RCT) [5] | 1.11 (0.62, 1.96) | -- |
| Rhythmicity (parent-rated STS at six to eight years) | | | |
| Baseline Hb > 110 g/L | | | |
| 20 mg oral iron versus placebo | 264 (1 RCT) [5] | -- | -0.10 (-0.29, 0.09) |
| Rhythmicity score >1 SD above mean (parent-rated STS at six to eight years) | | | |
| Baseline Hb > 110 g/L | | | |
| 20 mg oral iron versus placebo | 264 (1 RCT) [5] | 0.96 (0.57, 1.61) | -- |
| Inflexibility (parent-rated STS at six to eight years) | | | |
| Baseline Hb > 110 g/L | | | |
| 20 mg oral iron versus placebo | 264 (1 RCT) [5] | -- | -0.10 (-0.34, 0.14) |
| Inflexibility > 1 SD above mean (parent-rated STS at six to eight years) | | | |
| Baseline Hb > 110 g/L | | | |
| 20 mg oral iron versus placebo | 264 (1 RCT) [5] | 1.26 (0.73, 2.19) | -- |
| Persistence (parent-rated STS at six to eight years) | | | |
| Baseline Hb > 110 g/L | | | |
| 20 mg oral iron versus placebo | 264 (1 RCT) [5] | -- | 0.10 (-0.11, 0.31) |
| Persistence score > 1 SD above mean (parent-rated STS at six to eight years) | | | |

| | | | |
|---|---|---|---|
| Baseline Hb > 110 g/L | | | |
| 20 mg oral iron versus placebo | 264 (1 RCT) [5] | 1.53 (0.90, 2.58) | -- |
| Total easy-difficult temperament (parent-rated STS at six to eight years) | | | |
| Baseline Hb > 110 g/L | | | |
| 20 mg oral iron versus placebo | 264 (1 RCT) [5] | -- | 0.00 (-0.15, 0.15) |
| Total easy-difficult temperament score >1 SD above mean (parent-rated STS at six to eight years) | | | |
| Baseline Hb > 110 g/L | | | |
| 20 mg oral iron versus placebo | 264 (1 RCT) [5] | 1.15 (0.66, 1.99) | -- |
| **Prevention of IDA: other cognitive outcomes** | | | |
| Visual reasoning (SBIS at four years) | | | |
| Baseline Hb >110 g/L | | | |
| 20 mg oral iron versus placebo | 302 (1 RCT) [3] | -- | -1.00 (-3.37, 1.37) |
| Quantitative reasoning (SBIS at four years) | | | |
| Baseline Hb >110 g/L | | | |
| 20 mg oral iron versus placebo | 302 (1 RCT) [3] | -- | 0.00 (-2.71, 2.71) |
| Short-term memory (SBIS at four years) | | | |
| Baseline Hb >110 g/L | | | |
| 20 mg oral iron versus placebo | 302 (1 RCT) [3] | -- | 0.00 (-2.71, 2.71) |
| Fluid reasoning index (WPPSI-IV at four years) | | | |
| Baseline Hb 110-130 g/L | | | |
| 80 mg versus 40 mg oral iron | 182 (1 RCT) [4] | -- | -0.26 (-4.21, 3.69) |
| Baseline Hb > 130 g/L | | | |
| 40 mg versus 20 mg oral iron | 106 (1 RCT) [4] | -- | 2.34 (-2.46, 7.14) |
| Working memory index (WPPSI-IV at four years) | | | |
| Baseline Hb 110-130 g/L | | | |
| 80 mg versus 40 mg oral iron | 182 (1 RCT) [4] | -- | -0.29 (-3.82, 3.24) |
| Baseline Hb > 130 g/L | | | |
| 40 mg versus 20 mg oral iron | 106 (1 RCT) [4] | -- | -0.97 (-5.60, 3.66) |
| Processing speed index (WPPSI-IV at four years) | | | |

| | | | |
|---|---|---|---|
| Baseline Hb 110-130 g/L | | | |
| 80 mg versus 40 mg oral iron | 182 (1 RCT) [4] | -- | 1.85 (-1.92, 5.62) |
| Baseline Hb > 130 g/L | | | |
| 40 mg versus 20 mg oral iron | 106 (1 RCT) [4] | -- | 0.57 (-3.95, 5.09) |
| General ability index (WPPSI-IV at four years) | | | |
| Baseline Hb 110-130 g/L | | | |
| 80 mg versus 40 mg oral iron | 182 (1 RCT) [4] | -- | -0.06 (-3.67, 3.55) |
| Baseline Hb > 130 g/L | | | |
| 40 mg versus 20 mg oral iron | 106 (1 RCT) [4] | -- | 2.25 (-2.04, 6.54) |
| Cognitive proficiency index (WPPSI-IV at four years) | | | |
| Baseline Hb 110-130 g/L | | | |
| 80 mg versus 40 mg oral iron | 182 (1 RCT) [4] | -- | 1.59 (-1.99, 5.17) |
| Baseline Hb > 130 g/L | | | |
| 40 mg versus 20 mg oral iron | 106 (1 RCT) [4] | -- | 0.72 (-3.74, 5.18) |
| Visual-motor precision (NEPSY-II at four years) | | | |
| Baseline Hb 110-130 g/L | | | |
| 80 mg versus 40 mg oral iron | 182 (1 RCT) [4] | -- | 0.29 (-0.63, 1.21) |
| Baseline Hb > 130 g/L | | | |
| 40 mg versus 20 mg oral iron | 106 (1 RCT) [4] | -- | 0.92 (-0.26, 2.10) |

Abbreviations: ASQ AT 12 MONTHS: Ages and Stages Questionnaire; Bayley-III at 40 days: Bayley Scales of Infant Development version 3; Hb: haemoglobin; ID: iron deficiency; IDA: iron deficiency anaemia; IV FCM: intravenous ferric carboxymaltose; NEPSY-II: Neuropsychological Assessment second edition; SBIS: Stanford-Binet Intelligence Scale; SDQ: Strengths and Difficulties Questionnaire; STS: Short Temperament Scale; WPPSI-IV: Wechsler Preschool and Primary Scale of Intelligence version 4.

## References


1. Iglesias-Vazquez, L.; Hernandez-Martinez, C.; Voltas, N.; Canals, J.; Coronel, P.; Gimeno, M.; Arija, V. Adapting prenatal iron supplementation to maternal needs results in optimal child neurodevelopment: a follow-up of the ECLIPSES Study. *BMC Pregnancy Childbirth* **2022**, *22*, 710, doi:https://dx.doi.org/10.1186/s12884-022-05033-y.
2. Froessler, B.; Schubert, K.O.; Palm, P.; Church, R.; Aboustate, N.; Kelly, T.L.; Dekker, G.A.; Hodyl, N.A. Testing equivalence of two doses of intravenous iron to treat iron deficiency in pregnancy: A randomised controlled trial. *BJOG* **2023**, *130*, 15-23, doi:10.1111/1471-0528.17288.
3. Zhou, S.J.; Gibson, R.A.; Crowther, C.A.; Baghurst, P.; Makrides, M. Effect of iron supplementation during pregnancy on the intelligence quotient and behavior of children at 4 y of age: long-term follow-up of a randomized controlled trial. *Am J Clin Nutr* **2006**, *83*, 1112-1117.
4. Iglesias-Vazquez, L.; Voltas, N.; Hernandez-Martinez, C.; Canals, J.; Coronel, P.; Gimeno, M.; Basora, J.; Arija, V. Importance of Maternal Iron Status on the Improvement of Cognitive Function in Children After Prenatal Iron Supplementation. *Am J Prev Med* **2023**, https://dx.doi.org/10.1016/j.amepre.2023.02.006, doi:https://dx.doi.org/10.1016/j.amepre.2023.02.006.
5. Parsons, A.G.; Zhou, S.J.; Spurrier, N.J.; Makrides, M. Effect of iron supplementation during pregnancy on the behaviour of children at early school age: long-term follow-up of a randomised controlled trial. *Br J Nutr* **2008**, *99*, 1133-1139.
